# Impaired Performance on a Cognitively-Based Instrumental Activities of Daily Living Task, the 10-Item Weekly Calendar Planning Activity, in Individuals With Stroke Undergoing Acute Inpatient Rehabilitation

**DOI:** 10.3389/fneur.2021.704775

**Published:** 2021-07-21

**Authors:** Abhishek Jaywant, Catherine Arora, Alexis Lussier, Joan Toglia

**Affiliations:** ^1^Department of Psychiatry, Weill Cornell Medicine, New York, NY, United States; ^2^Department of Rehabilitation Medicine, Weill Cornell Medicine, New York, NY, United States; ^3^NewYork-Presbyterian Hospital/Weill Cornell Medical Center, New York, NY, United States; ^4^School of Health and Natural Science, Mercy College, Dobbs Ferry, NY, United States

**Keywords:** neurorehabilitation, executive functioning, activities of daily living, cerebrovascular disease, neuropsychology

## Abstract

Performance-based, functionally relevant, and standardized measures of cognitive-instrumental activities of daily living (C-IADL) can complement neuropsychological tests of cognitive impairment and provide valuable clinical information to inform rehabilitation planning. Existing measures have been validated in the outpatient setting. Here, we sought to evaluate a 10-item, short-form of a C-IADL measure, Weekly Calendar Planning Activity (WCPA-10), in inpatients with stroke undergoing acute rehabilitation. The specific goal was to determine if the WCPA-10 could differentiate between stroke patients undergoing acute inpatient rehabilitation and healthy control individuals. We also explored whether the WCPA-10 would identify C-IADL limitations in stroke patients screened as having intact cognition. Seventy-seven stroke inpatients undergoing rehabilitation and 77 healthy control participants completed the WCPA-10, which involves entering a list of simulated, fictional appointments into a weekly schedule while keeping track of and adhering to multiple task rules and ignoring built-in obstacles and distractions. Compared to the control group, stroke patients had significantly worse accuracy, made more errors, used fewer cognitive strategies, followed fewer rules, took more time to complete the task, and were less efficient. 83% of stroke patients were less accurate than predicted by their age, and 64% used less strategies than their age prediction. Among 28 participants who screened as having “normal” cognitive function on the Montreal Cognitive Assessment, the majority had deficits on the WCPA-10. Our results provide initial support for use of a brief C-IADL assessment, WCPA-10, for individuals with stroke undergoing inpatient rehabilitation. They indicate that stroke patients have deficits in C-IADL accuracy, efficiency, and strategy use at this stage of stroke recovery. Results highlight the need to use performance based, functional cognitive assessments, even for those who perform well on cognitive screening tools.

## Introduction

Cognitive impairments are common and persistent following stroke and contribute to limitations in daily activities and poor functional outcomes (1). Neuropsychological testing is the gold standard for assessing cognition in stroke patients at the impairment level. Functional cognitive assessments that objectively assess performance in complex or cognitively-based instrumental activities of daily (C-IADL)— such as organizing a schedule, paying bills, or managing medications—can serve as a valuable complement when assessing cognition in stroke patients. C-IADL measures reflect the integration of multiple cognitive skills, predominantly executive functions, applied to functionally relevant activities (2). An individual with stroke may perform well on structured neuropsychological measures, but have considerable difficulty in everyday unstructured activities that require the ability to initiate, plan, multitask or cope with unexpected obstacles. Although performance on C-IADL tasks is associated with standardized neuropsychological tests, the correlations only range between 0.27 and 0.60, suggesting that each provides unique contributions to characterizing the person's overall cognitive profile (3).

C-IADL measures can be particularly valuable for stroke patients in the acute inpatient rehabilitation setting, because they can identify functional cognitive weaknesses and inform early cognitive rehabilitation intervention. This is important because early post-stroke executive dysfunction is associated with long-term disability and limitations in activities of daily living (4–6). Cognitive difficulties in the early post-stroke period are also independently associated with functional mobility in the chronic phase (7), possibly because impaired cognition interferes with attention to and control of motor movements (8), particularly when the difficulty of walking is high (9). Early, tailored cognitive interventions can alter the trajectory of recovery post-stroke (10). C-IADL measures may also be optimal for administration during acute inpatient rehabilitation because they are within the scope of practice of occupational therapists, and do not require specialty consultation with a neuropsychologist.

There are few performance-based C-IADL assessments that have been described specifically for the inpatient rehabilitation of stroke patients. Exceptions include the Executive Function Performance Test, which incorporates bill paying, medication management, using the telephone, and cooking (11); and the Kettle Test, which involves preparing beverages according to specific criteria (12). Both measure the level of verbal assistance needed to complete the task; however, feasibility can be constrained by the kitchen and cooking equipment needed for the Kettle Test and the cooking subtests of the Executive Function Performance Test. The Multiple Errands Test (13) is another real-world measure of executive function, for which an inpatient, hospital-based version has been developed (14). It requires multitasking and suppression of habitual responses, similar to ecologically-valid measures of executive functions that were previously developed for adults with brain injuries such as the Six Elements Test (15) and the Hotel task (16). A limitation of the hospital-based Multiple Errands Test is that it is site-specific and requires patients to be moved off unit to the hospital lobby, which can reduce feasibility given the time constraints of the inpatient setting.

The Weekly Calendar Activity (WCPA) (17, 18) is a complex C-IADL measure, similar to the MET, that can be implemented on a desktop or table using only paper and pencil. It involves entering a list of simulated, fictional appointments into a weekly schedule while keeping track of and adhering to multiple rules. Some appointments have set days and times (“fixed”) while others include choices of days or times (“flexible”) so the person has to make decisions, plan ahead and problem-solve to manage potential conflicts. The task of entering appointments into a weekly schedule is easily recognized as relevant to functional abilities in everyday life and appears easy on the surface; however, appointment conflicts, rule constraints and unexpected obstacles create significant cognitive challenges that require a strategic approach. The standard 17-item version of the WCPA differentiates between healthy controls and a wide range of populations with executive dysfunction including those with multiple sclerosis (19), mild cognitive impairment (20), attention-deficit hyperactivity disorder (21), pediatric acquired brain injury (22), and epilepsy (23). Accuracy on the WCPA correlates with inhibitory control and set-shifting as assessed by the Delis-Kaplan Executive Functioning System (19).

A shorter 10 item version of the WCPA (WCPA-10) was created to decrease the time needed for administration and to be more feasible for the inpatient setting. Seven of the easiest items from the WCPA-17 item appointment list were removed. All other components remained exactly the same. Whether the WCPA-10 can differentiate between healthy adults and individuals with stroke in evaluating C-IADL ability after stroke, and specifically in the inpatient rehabilitation setting with the shorter 10-item version, has to date not been established. Given that the WCPA-10 relies on planning, working memory shifting, and inhibition—abilities that are frequently impaired post-stroke (24, 25)—the WCPA may be sensitive to C-IADL deficits and differentiate patients from age-matched healthy adults in the acute inpatient rehabilitation setting.

The goal of this study was to compare individuals with stroke to healthy age-matched adults in performance on the 10-item short-form/inpatient version of the WCPA. We hypothesized that relative to the healthy control group, individuals with stroke would have lower percentage accuracy of appointments entered, and a lower number of strategies used, which are the primary outcomes of C-IADL and cognitive strategy use, respectively, on the WCPA-10. We also hypothesized that compared to healthy participants, stroke patients would spend less time planning, take longer to complete the task, follow fewer rules correctly, and use fewer cognitive strategies. We predicted that WCPA-10 performance would be correlated only modestly with an impairment-level screening measure of cognition, given that there is only partial overlap between impairment-based and C-IADL measures of cognition (26). Finally, we explored whether the WCPA-10 would be sensitive to C-IADL dysfunction in individuals who screened as having normal cognitive functioning.

## Methods and Materials

### Participants

*N* = 77 individuals with stroke and *N* = 77 healthy age matched controls from a larger existing normative database were included in this study. Stroke patients were all undergoing acute inpatient rehabilitation on a 22-bed general rehabilitation unit at a large, urban academic medical center. Inclusion criteria were the same as for admission to the inpatient rehabilitation unit: medically stable for rehabilitation, ability to tolerate 3 h of rehabilitation therapy daily, and reasonable expectation for functional gain. The 10-item short form of the WCPA was administered to accommodate the time constraints of the inpatient setting. The WCPA-10 was administered as part of standard of care on the inpatient rehabilitation unit by Occupational Therapists for persons who were alert, oriented, able to attend for at least 20 min, able to read and write legibly in English, follow two-step commands, and were cognitively independent in basic self-care activities of daily living (ADL). Exclusion criteria included those who would not be typically given the WCPA-10 during ordinary care such as those with dementia, severe cognitive impairment, language or visual deficits, or required cognitive assistance for basic self-care activities. People with limited English proficiency were also excluded as the test materials were only available in English. All study procedures were approved by the Weill Cornell Medicine Institutional Review Board.

Healthy control participants were obtained from an existing normative database. Participants were recruited *via* snowballing techniques by graduate occupational therapy students from the greater New York City area. Inclusion criteria were those who were living independently in the community, and for participants age over 65, a score >24 on the Montreal Cognitive Assessment (when available, conducted in 46/77 participants). Exclusion criteria were subjective cognitive complaints as measured by a standardized T-score < 35 on the Patient-Reported Outcomes Measurement Information System, (PROMIS) Cognitive Abilities Short-Form Version 2.0, Form 8a (27); reported past history of a neurological condition (e.g., previous stroke, traumatic brain injury, Parkinson's disease, brain tumor), attention-deficit hyperactivity disorder, history of hospitalization for a psychiatric disorder, or inability to read or write in English. Collection of normative data from healthy controls was granted exemption by the Mercy College Institutional Review Board (IRB), because data were recorded such that participants could not be identified. An oral consent script was read aloud, and a written copy of the script was provided to each participant.

### Measures

#### 10-item Weekly Calendar Planning Activity

**Figure 1:**
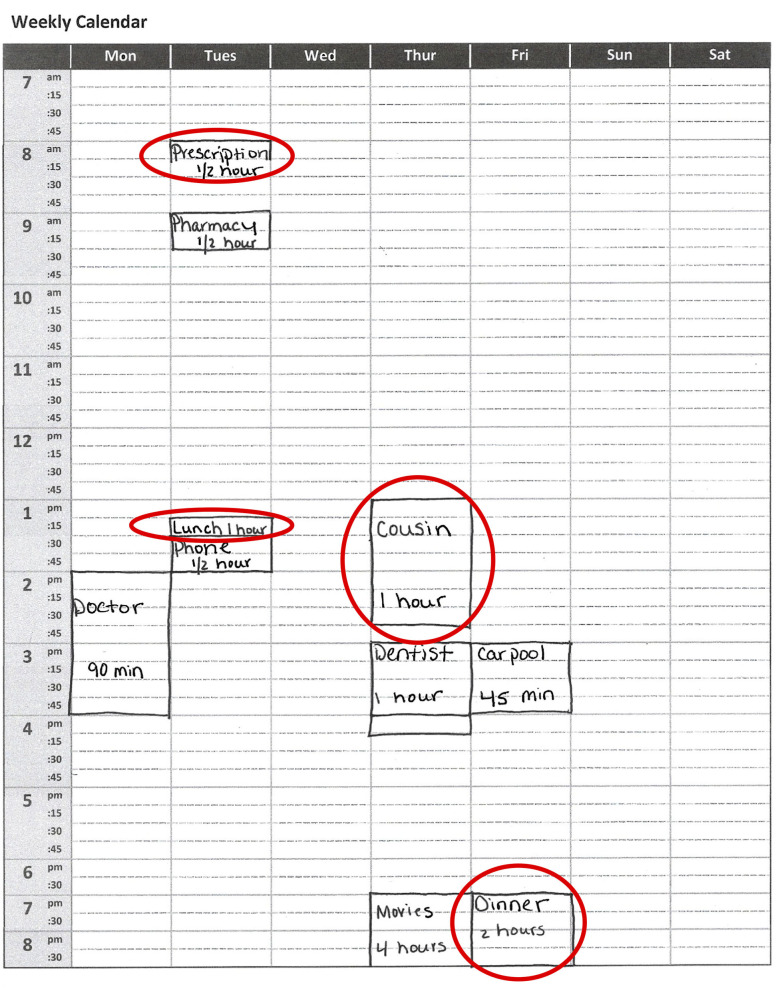
Visual example of the weekly calendar stimulus on the Weekly Calendar Planning Activity. Patients are required to schedule appointments of specific lengths on specific days and times while following multiple rules. Red circles highlight errors, which can include placing the appointment on the wrong day or time (“Prescription ½ h”); marking the appointment with an incorrect duration (“Lunch 1 h”; “Cousin 1 h”); or having a vague description of the appointment (“Cousin,” “Dinner”).

The WCPA-10 is an objective measure of C-IADL performance. The original 17 item version has demonstrated validity, reliability, and sensitivity to executive dysfunction and sensitivity to change (17, 18, 28, 29). The WCPA-10 requires the examinee to input a series of appointments into a mock weekly calendar/schedule while following a set of specific rules and guidelines (Figure 1). Appointments are either fixed at a certain date/time or flexible and can be entered on multiple dates/times, and at times conflict, which requires the examinee to manage conflicting appointments. The examinee has to keep track of multiple rules (e.g., cannot enter appointments on a certain day, cannot cross off items once entered) in working memory while shifting between the appointment sheet, calendar, and instructions sheets. The rules are explained verbally just prior to beginning the task. An 8 × 11 paper with task instructions is also placed on the table and can be referred to by the examinee throughout the task. The examiner periodically attempts to distract the examinee with pre-specified questions, which the examinee has to inhibit. The examiner observes the examinee and records specific strategies that he or she uses; the examinee also reports to the examiner at the end of the task any additional strategies that he or she employed in a post-task interview.

In this study, we used the 10-appointment version of the WCPA. The WCPA-10 has the same ratio of fixed and variable appointments (3/7 or 70%) as the original WCPA, but there are only 10 appointments to enter as opposed to 17. The main outcome measure was the percent of appointments entered correctly out of 10 (Percent Accuracy, i.e., number correct/10 × 100%), as it incorporates both accurate performance, errors in managing conflicts, and omission errors. Total Strategies (combination of those observed by the examiner and self-reported by the examinee) was a second measure emphasized in analyses, given the importance of cognitive strategies to cognitive rehabilitation. We also calculated Planning Time (time in seconds from the start of the task to entering the first appointment), Time to Completion, Efficiency Score (time in seconds/weighted accuracy), Total Errors, and the number of Rules Followed correctly out of 5. A lower efficiency score indicates that the client obtained higher accuracy in less time. Efficiency scores were not calculated for those with accuracy scores of 3 or below. Based on the standard WCPA-10 record form, we also documented for each participant whether or not they used one of several different cognitive strategies. Finally, at the conclusion of the WCPA-10, participants were asked “Do you do tasks like this on a regular basis?” to gauge their familiarity and responded “yes” or “no.”

#### Montreal Cognitive Assessment

The MoCA (30) is a 30-item screening measure for general cognitive impairment that is administered on admission as standard of care to all stroke patients on our acute inpatient rehabilitation unit. The MoCA assesses visuospatial/executive skills, naming, attention, language, abstraction, delayed recall, and orientation. Lower scores indicate greater cognitive impairment. The MoCA has demonstrated validity and clinical utility in inpatient stroke rehabilitation (31), and is closely associated with impairments assessed using neuropsychological tests (4).

### Statistical Analysis

We used one way analysis of variance (ANOVA) and chi-square tests to evaluate group differences in demographic and clinical variables. We used one-way ANOVAs to evaluate group differences on each of the outcome measures, Percent Accuracy, Planning Time, Time to Completion, Efficiency Score, Total Strategies, Total Errors, and Total Rules Followed. Although all WCPA-10 variables differed from normality using the Kolmogorov-Smirnov test (all *p*'s < 0.01), ANOVA is known to be robust against violations of normality (32). The use of non-parametric tests did not change any findings; thus, we report ANOVA results.

For cognitive strategies that were commonly used by the healthy control group (at least *n* = 20 [25%] of the control group used), we compared the frequency of use by individuals with stroke to healthy control participants using chi-square tests. We used a chi-square test to compare the frequency of yes vs. no vs. missing responses to familiarity question by group, and then an independent samples *t*-test to evaluate separately in stroke and control participants whether there was a difference in accuracy by familiarity (yes or no). We evaluated the association between cognitive impairment and WCPA-10 performance separately in stroke and healthy participants using Spearman rank-order correlations between MoCA scores and Percent Accuracy, Planning Time, Time to Completion, Efficiency Score, Total Strategies, and Total Rules Followed.

We next sought to explore individual differences in the performance of stroke participants relative to the healthy control group, correcting for demographic factors. We first used Spearman rank-order correlations to evaluate in the healthy control group the association between age and education, with Percent Accuracy (as a measure of overall executive skills) and Total Strategies (as a measure of cognitive strategy use). We then used demographic-corrected regression equations—including predictors that exhibited significant correlations with Percent Accuracy and Total Strategies—to obtain the demographic-predicted score for each stroke participant. We subsequently subtracted each participant's demographic-predicted score from his or her obtained score to obtain the residual demographic-corrected score. We reported the frequencies of these residual scores for the entire sample, and for those patients who scored in the normal range on the MoCA (25 or greater out of 30), the latter in order to explore the clinical utility of the WCPA-10 in individuals with stroke who screen as having normal cognitive functioning.

## Results

### Demographics and Clinical Characteristics

There were no group differences in age, gender, or education (Table 1). There was a significant difference in race/ethnicity between groups. Both groups had similar percentages of Caucasian participants, while a greater percentage of Black participants and a smaller percentage of Hispanic participants were observed in the stroke group. Stroke participants had significantly lower MoCA scores than the healthy control group.

**Table 1:** Demographic and clinical characteristics.

	**Stroke (*N* = 77)**	**Healthy control (*N* = 77)**	***F*-value**	**df**	***p*-value**	**Effect size** **η**^**2**^
Age	66.1 (14.1)	66.0 (14.0)	0.00	1,152	0.99	0.00
Gender	Female: 38 (49%) Male: 39 (51%)	Female: 41 (53%)Male: 36 (47%)			0.63	–
Education (years)	14.7 (1.9)	14.6 (2.6)	0.04	1,119	0.85	0.00
Race/ethnicity	White: 51 (67%) Black: 16 (21%) Hispanic: 3 (4%) Asian/Pacific Islander: 4 (5%) Native American: 0 (0%) Other: 2 (3%)	White: 49 (64%)Black: 11 (14%)Hispanic: 15 (20%)Asian/Pacific Islander: 1 (1%)Native American: 1 (1%)Other: 0 (0%)			0.02	–
Stroke location	Right hemisphere: 38 (49%) Left hemisphere: 28 (36%) Bilateral: 8 (10%) Unknown/not available: 3 (4%)					
Days post-stroke	18.1 (14.6)	–			–	
Montreal cognitive assessment	23.3 (3.6)	26.2 (1.7)	26.1	1,121	<0.001	0.18

### Performance on the WCPA-10

On average, the WCPA-10 took ~12–13 min for stroke participants to complete. Using one-way ANOVAs, relative to control participants, stroke patients had significantly worse Percent Accuracy, Total Strategies, Time to Completion, Efficiency Score, Rules Followed, and Total Errors (Table 2). Stroke patients and control participants did not differ in WCPA-10 Planning Time.

**Table 2:** Performance of stroke and healthy participants on the WCPA-10.

**WCPA-10 measure**	**Stroke**			**Healthy control**	***F*-value**	**df**	***p*-value**	**Effect size** **η**^**2**^
	All cases	Low MoCA (<25)	High MoCA (≥25)					
Percent accuracy	49.9 (24.1)	45.7 (24.0)	57.1 (22.9)	71.0 (18.6)	37.2	1, 152	<0.001	0.20
Total strategies	3.9 (2.0)	3.5 (1.8)	4.6 (2.2)	5.0 (2.5)	10.2	1, 149	<0.002	0.06
Planning time (s)	89.5 (199.1)	79.8 (128.5)	106.1 (285.1)	62.9 (75.5)	1.2	1, 141	0.29	0.01
Time to completion (s)	767.1 (399.6)	805.4 (399.4)	699.0 (398.3)	552.8 (196.9)	17.7	1, 150	<0.001	0.11
Efficiency score	266.7 (242.3)	316.4 (283.7)	198.6 (150.5)	120.1 (72.1)	24.4	1, 129	<0.001	0.16
Rules followed	3.7 (1.0)	3.4 (1.0)	4.0 (0.8)	4.2 (0.8)	13.3	1, 149	<0.001	0.08
Total errors	5.0 (2.4)	5.4 (2.4)	4.3 (2.3)	2.9 (1.9)	37.2	1, 152	<0.001	0.20

*The statistics provided are for the comparison between all stroke cases and healthy control participants. For the measure Efficiency Score, higher scores indicate lower efficiency*.

**Figure 2:**
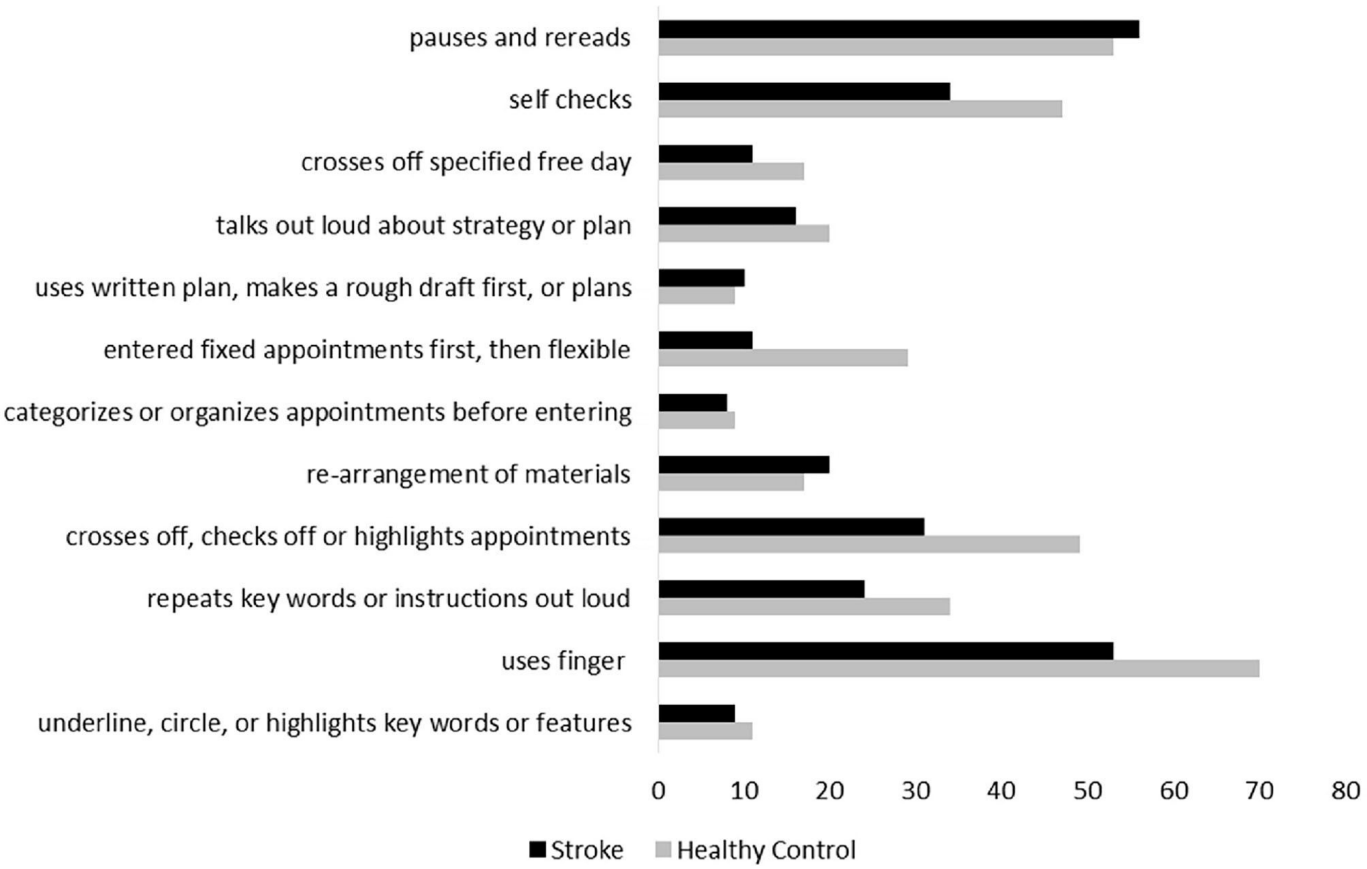
Frequency of strategies used by stroke patients and healthy control participants.

The number of strategies used was significantly related to the percentage of accurate appointments on the WCPA-10 (*r*_*s*_ = 0.37, *p* <0.001). The following strategies were used by at least *n* = 20 (25%) of the healthy control group: repeats keywords or instructions out loud; uses finger; crosses off, checks off, or highlights appointments entered; enters fixed appointments first and then flexible appointments; self-checks; talks out loud about strategy or plan; and pauses and rereads. Individuals with stroke less frequently used their finger, crossed/checked/highlighted appointments, entered fixed appointments first and then flexible appointments, and self-checked (Figure 2; all *X*^2^ > 8.1, *p*'s < 0.04). There was no group difference in frequency of repeating keywords/instructions out loud, or in frequency of pausing and rereading. A chi-square test comparing familiarity with a calendar/schedule format by group was significant [$X_{\left(2\right)}^{2}$ > 7.9, *p* = 0.02]; however, a z-test comparing cell proportions did not indicate a statistically significant difference in the proportion of the stroke group who stated they were familiar with the calendar (53%) vs. the control group (64%). In the stroke group, there was no difference between those who said they regularly used a calendar/schedule vs. those who said they did not in Percent Accuracy [*t*_(73)_ = 0.93, *p* = 0.36] or Total Strategies [*t*_(70)_ = 1.45, *p* = 0.15]. In the control group, there was no difference between those who said they regularly used a calendar/schedule vs. those who said they did not in Percent Accuracy [*t*_(67)_ = 1.48, *p* = 0.15] or Total Strategies [*t*_(67)_ = 0.27, *p* = 0.79].

### Correlation With Cognitive Impairment

In stroke participants, performance on the MoCA was modestly but significantly correlated with Percent Accuracy (*r*_s_ = 0.31, *p* = 0.006), Rules Followed (*r*_s_ = 0.31, *p* = 0.007), and Total Strategies (*r*_s_ = 0.30, *p* = 0.009). MoCA score was not correlated with Efficiency Score (*r*_s_ = −0.25, *p* = 0.06), Time to Completion (*r*_s_ = –.08, *p* = 0.49) or Planning Time (*r*_s_ = −0.08, *p* = 0.53). In healthy participants, performance on the MoCA was modestly but significantly correlated with Total Strategies (*r*_s_ = 0.40, *p* = 0.006), but not Percent Accuracy (*r*_s_ = 0.14, *p* = 0.34), Rules Followed (*r*_s_ = −0.03, *p* = 0.84), Efficiency Score (*r*_s_ = 0.11, *p* = 0.50), Time to Completion (*r*_s_ = 0.27, *p* = 0.07) or Planning Time (*r*_s_ = 0.23, *p* = 0.13).

### Exploratory Evaluation of Individual Differences in Performance in Stroke Participants Relative to Control Group After Demographic Correction

In the healthy control group, Percent Accuracy correlated significantly with age (*r*_s_ = −0.38, *p* < 0.001) but not education (*r*_s_ = 0.16, *p* = 0.19). Similarly, Total Strategies correlated significantly with age (*r*_s_ = −0.51, *p* < 0.001) but not education (*r*_s_ = 0.15, *p* = 0.21). We thus computed regression equations predicting Percent Accuracy and Total Strategies from age. The relationship between Percent Accuracy and age was modeled by *y* = 106.3 + (−0.53)^*^(age), and the relationship between Total Strategies and age was modeled by *y* = 10.5 + (−0.08)^*^(age). Using these equations, we calculated each stroke participant's age-predicted Percent Accuracy score and Total Strategies score, and subtracted these values from their obtained scores.

**Figure 3:**
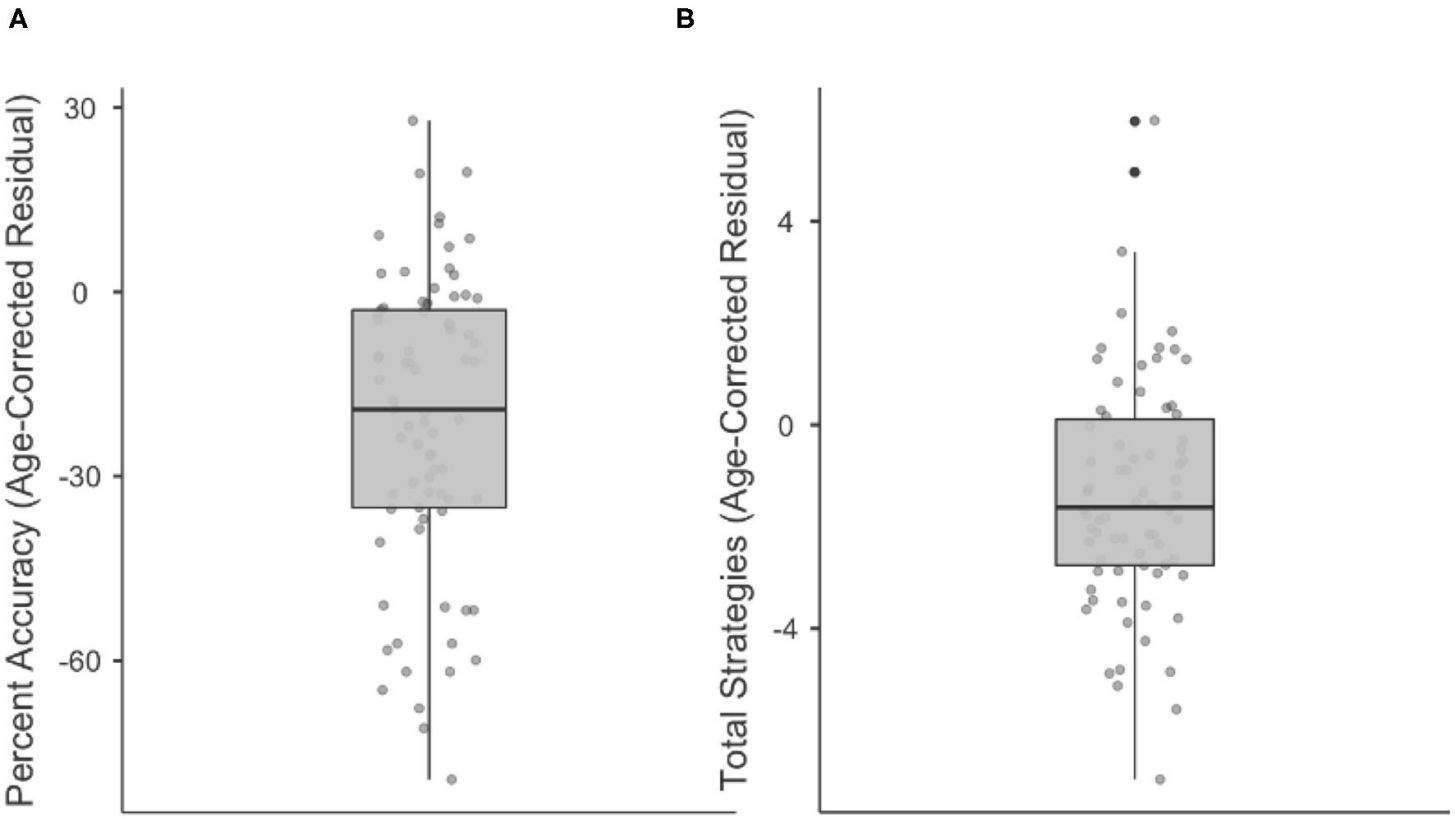
Boxplots showing median, interquartile range, range, and individual datapoints of stroke patient residual scores (raw score—age-predicted score) for percent accuracy **(A)** and total strategies **(B)**. Median/interquartile range of residual scores are below age predictions.

Results are displayed as box plots (median and interquartile range) in Figure 3, with negative values indicating performance worse than would be expected by age. As a group, stroke participants had a median Percent Accuracy 19.1% lower than would be predicted by age (range = 79.4% lower to 27.9% higher). 64/77 (83.1%) stroke participants were less accurate on the WCPA than their age prediction. Similarly, stroke participants as a group had a median Total Strategies 1.6 lower than would be predicted by age (range = 7 lower to 6 higher). 55/74 (74%) stroke participants used fewer strategies than their age prediction; three stroke participants were missing data on strategy use.

**Figure 4:**
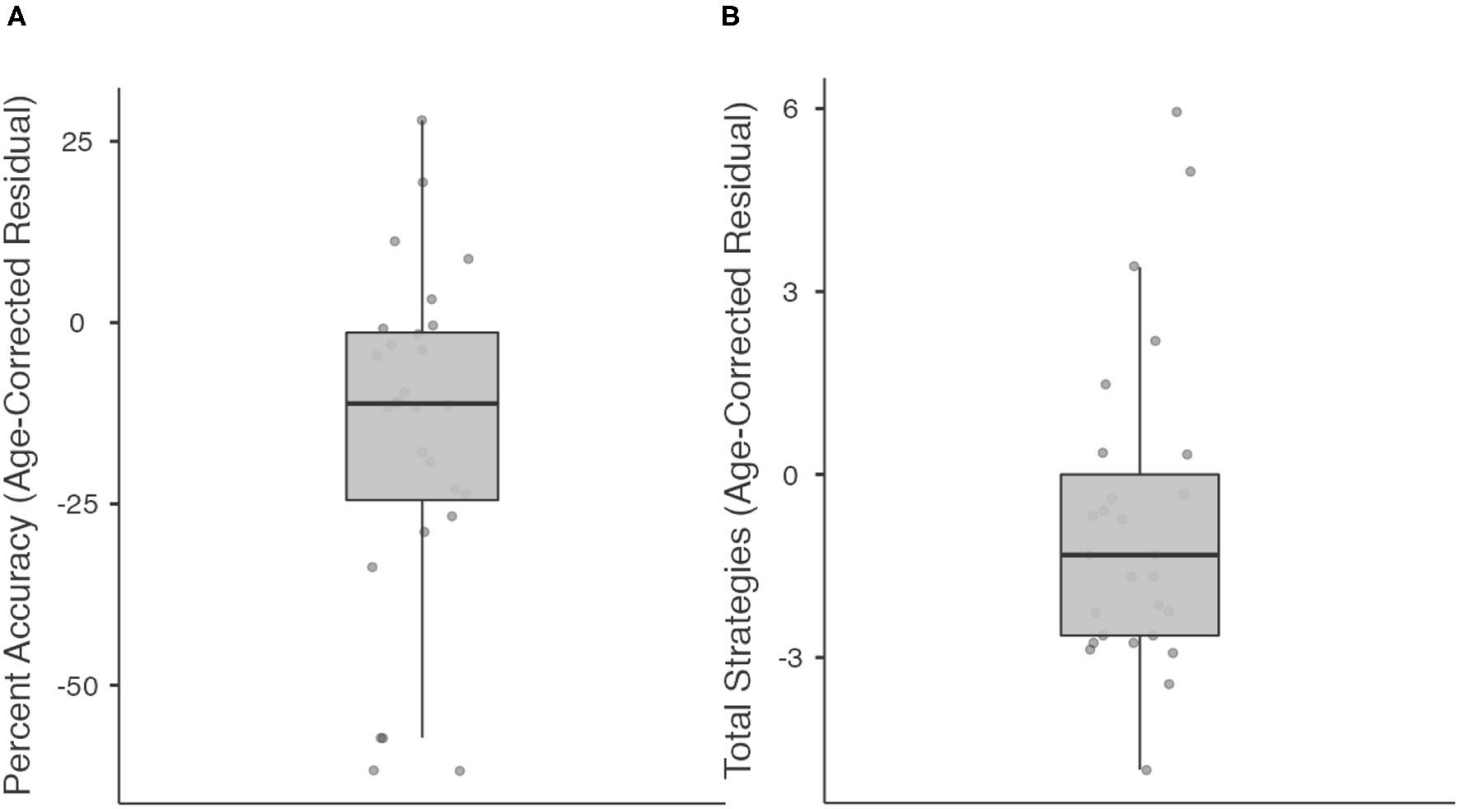
Boxplots showing median, interquartile range, range, and individual datapoints of stroke patient residual scores (raw score—age-predicted score) for percent accuracy **(A)** and total strategies **(B)**, in patients deemed to have “normal” cognitive function on the Montreal Cognitive Assessment. Median/interquartile range of residual scores are below age predictions.

We then explored individual differences in performance (Percent Accuracy and Total Strategies) using the regression-predicted and age-corrected procedure above, but in stroke participants who scored within normal limits (25/30 or higher) on the MoCA (Figure 4). Such participants would be classified clinically as having “normal” cognitive functioning based on standard of care cognitive screening on our inpatient rehabilitation unit. Twenty-eight individuals in our sample scored within normal limits on the MoCA. Within this subgroup, median Percent Accuracy was 11.2% lower than age prediction (range: 61.8% lower to 27.9% higher). 23/28 stroke participants (82.1%) performed below their age-predicted score in Percent Accuracy. Within this subgroup, median Total Strategies was 1.32 lower than predicted by age (range: 4.8 lower to 6 higher). 20/27 stroke participants (74.1%; 1 individual with missing data) performed below their age-predicted score in Total Strategies.

## Discussion

The results of this study provide initial support for use of a brief C-IADL assessment,–the WCPA-10–for individuals with stroke undergoing inpatient rehabilitation and highlight the need to use performance based, functional cognitive assessments, even for those who perform well on cognitive screening tools. Specifically, we found that our stroke sample exhibited greater C-IADL deficits, and used fewer cognitive strategies, than did healthy control participants. At an individual level, the majority of stroke patients score below their age-predicted performance on the WCPA-10, including overall accuracy and total strategies used. Performance on the WCPA-10 correlated only modestly with an impairment-based screening measure of cognition (MoCA) and identified deficits in patients who would be deemed to have “normal” cognition based on the MoCA.

The WCPA-10 differentiated individuals with stroke from healthy control participants on multiple aspects of C-IADL performance and identified performance deficits that can be easily missed within a structured inpatient rehabilitation setting. Specifically, relative to the control group, our sample of stroke patients had significantly lower accuracy, followed fewer rules, made a greater number of errors, were less efficient, and took longer to complete the WCPA-10. At an individual level, use age-the majority of stroke patients (83%) performed worse on the WCPA-10 than their age prediction. Further, the majority of stroke patients (74%) used fewer cognitive strategies than their age prediction. Because we did not have a detailed cognitive assessment to which we could compare WCPA-10 performance, the specific cognitive impairments contributing to deficient performance are unknown. However, prior research has demonstrated an association between the 17-item WCPA and executive functions (17, 19, 20), suggesting that executive dysfunction may have impacted performance.

Importantly, the WCPA-10 identified C-IADL deficits and worse cognitive strategy use in patients who scored within the normal range on the MoCA. Eighty-two percentage of patients classified as “normal” on the MoCA had worse accuracy than their age prediction and 74% used fewer strategies than their age prediction. This finding underscores the utility of a C-IADL measure such as the WCPA-10 as a complement to traditional impairment-based cognitive screening measures such as the MoCA. Put another way, relying solely on a screen such as the MoCA may result in missing cognitive limitations that have the potential to impact patients' independence in daily activities. Given that it can be administered in on average 12 min, the WCPA-10 can complement the MoCA to assist in identifying and triaging patients most in need of follow-up comprehensive neuropsychological evaluation or higher level functional testing, which can provide information on specific underlying cognitive impairments that may be impacting functional performance. Relatedly, we found only modest correlations between the WCPA and the MoCA. This finding accords with research indicating only partial overlap between impairment-based and functional measures of cognition (26, 33).

Interestingly, the stroke and control groups did not differ in planning time on the WCPA-10. That is, stroke patients on average did not take more or less time relative to control participants to plan their approach to the task, prior to initiating the first appointment entered. This may be because the WCPA-10 goal of entering a list of appointments into a calendar appears deceptively easier than it actually is. Healthy control participants also demonstrated relatively brief planning times; however, they were observed to more frequently stop, pause and readjust task methods once they encountered potential appointment conflicts or recognized task complexities. Pause and stop periods within the task, may thus be better indications of planning than the initial planning time in this particular task.

An advantage of the WCPA-10 is that it enables the objective quantification of cognitive strategy use. This is especially relevant in the inpatient rehabilitation setting where rehabilitation clinicians are teaching patients strategies to optimize performance and maximize independence in C-IADLs in preparation for discharge back to the community. Cognitive strategies are normally used to help people monitor and control performance errors or manage task challenges in cognitively demanding tasks. Healthy people typically use multiple strategies when faced with a cognitive challenge and this was observed with healthy controls on the WCPA. Our findings suggest deficiencies in cognitive strategy use and is consistent with other literature reporting decreases in cognitive strategy use in people with acquired brain injury (34). We found that individuals with stroke less frequently used particular types of cognitive strategies on the WCPA-10. Specifically, they less frequently used their finger (i.e., to focus and maintain attention on salient aspects of the stimuli), less frequently crossed out/checked off/highlighted appointments to keep track of those that had been entered and those that had not been entered, less often entered fixed appointments first and then flexible appointments, and less frequently self-checked for errors. The lower use of these strategies may have increased demands on working memory and cognitive load, thereby contributing to worse performance. This is consistent with studies on the association between strategies and functional performance (34–36). Decreased self-awareness of performance may also be a factor contributing to decreased strategy use (37). For example, if a person doesn't recognize challenges or task difficulties, they also may not perceive the need to use strategies. Future research is needed to examine the cognitive strategy score on the WCPA-10 and its relationship to self-awareness.

Careful analysis of performance and strategy deficiencies within the context of the WCPA-10 can inform the types of strategies and training that may be most useful for clinicians to emphasize during rehabilitation. The WCPA-10 identifies people who have difficulty managing a list and entering information accurately into a weekly calendar. Since use of lists and schedules is an inherent aspect of many everyday tasks, identification of difficulties in these areas provides important targets for rehabilitation intervention. For example, functional cognitive rehabilitation activities that involve managing use of lists in a wide variety of contexts have been described by others (28, 38). The WCPA-10 may also provide more general information on underlying performance deficits, error patterns and deficiencies in strategies that are likely to influence functioning across multiple step activities. Different WCPA-10 result patterns can be observed by analyzing the combination and type of rule breaks, error types, efficiency, strategies used and responses to the after-task interview, along with accuracy. This is illustrated in the original WCPA test manual (17). For example, a person that omits appointments from the list, loses track of rules, and does not to check off appointments or self-check work might also show similar performance errors across other multiple step activities. Cognitive rehabilitation might address general methods to help the person initiate, manage and use efficient strategies to increase the ability to keep track of task variables.

### Limitations

Our characterization of clinical stroke characteristics was relatively limited. Because our data were collected in the context of routine clinical care, this limited the ability to collect more comprehensive information such as stroke location or type, lesion size, stroke severity, or medical comorbidities. However, this reflects the realities of clinical research in an acute inpatient rehabilitation setting. Future work on the WCPA-10 will benefit from investigating the relationship between clinical-disease characteristics and performance. Relatedly, the MoCA is a relatively brief screening measure of cognitive impairment. Our stroke sample was not routinely administered comprehensive neuropsychological measures of executive functioning and other cognitive domains to which we could compare performance on the WCPA-10. However, this reflects the reality of integrating assessments on acute inpatient rehabilitation units in which it is not always feasible to conduct extensive neuropsychological testing.

### Conclusion

The WCPA-10, a multi-step functional cognitive (C-IADL) task is feasible in an inpatient setting, relatively quick to administer, and captures functional performance deficits in stroke patients relative to age-matched healthy adults, even in those who perform above the normal cut-off score on a cognitive screening tool (Montreal Cognitive Assessment). Relative to healthy adults, individuals with stroke, also use significantly fewer cognitive strategies, both at the group level and commonly on an individual level. This finding emphasizes the importance of analyzing deficiencies in cognitive strategy use and considering methods for promoting strategy use within rehabilitation. C-IADL skills are typically under-assessed in inpatient rehabilitation settings in people with stroke due to time constraints and a focus on physical abilities and self-care skills. This paper is the first to report findings of the 10-item version of the WCPA, thereby contributing to the limited literature on C-IADL assessment and strategy use in stroke inpatients undergoing rehabilitation. The results highlight the potential utility of a higher-level functional cognitive assessment tool like the WCPA-10 to identify cognitive difficulties that may interfere with safety and independence upon discharge to the home and community.

## Data Availability Statement

The raw data supporting the conclusions of this article will be made available by the authors, without undue reservation.

## Ethics Statement

The studies involving human participants were reviewed and approved by Weill Cornell Medicine Institutional Review Board (stroke) and Mercy College (healthy participants). Stroke patients provided their written informed consent and Healthy participants provided oral consent.

## Author Contributions

AJ: conceptualization, formal analysis, writing-original draft, writing-review and editing, and visualization. CA: data curation, formal analysis, and writing-review and editing. AL: writing-review and editing and visualization. JT: conceptualization, methodology, writing-original draft, writing-review and editing, supervision, and project administration. All authors contributed to the article and approved the submitted version.

## Conflict of Interest

JT is author of the Weekly Calendar Planning Activity, published by AOTA Press and receives royalties for this publication. The remaining authors declare that the research was conducted in the absence of any commercial or financial relationships that could be construed as a potential conflict of interest.
